# Impact of herbivory and competition on lake ecosystem structure: underwater experimental manipulation

**DOI:** 10.1038/s41598-018-30598-0

**Published:** 2018-08-14

**Authors:** Ivana Vejříková, Lukáš Vejřík, Jan Lepš, Luboš Kočvara, Zuzana Sajdlová, Martina Čtvrtlíková, Jiří Peterka

**Affiliations:** 1 Biology Centre of the Czech Academy of Sciences, Institute of Hydrobiology, Na Sádkách 7, 37005 České Budějovice, Czech Republic; 2 Biology Centre of the Czech Academy of Sciences, SoWa, Na Sádkách 7, 37005 České Budějovice, Czech Republic; 3 University of South Bohemia in České Budějovice, Faculty of Science, Branišovská 31, 37005 České Budějovice, Czech Republic

## Abstract

Two basic ecological relationships, herbivory and competition, distinctively influence terrestrial ecosystem characteristics, such as plant cover, species richness and species composition. We conducted a cage experiment under natural conditions in an aquatic ecosystem to test the impacts of two treatments combined in a factorial manner: (i) a pulse treatment – removal of dominant competitors among primary producers (macroalgae *Chara* sp. and *Vaucheria* sp.), and (ii) a press treatment – preventing herbivore (fish, crayfish) access to caged plots. The plots were sampled once before the treatments were established and four more times within two years. Both treatments had a significantly positive impact on macrophyte cover and species richness and changed the macrophyte species composition. The effect of the macroalgae removal was immediate with the highest species richness occurrence during the first post-treatment monitoring, but the positive effect vanished with time. In contrast, preventing herbivore access had a gradual but long-lasting effect and reached a more steady-state over time. Two of the most common species showed contrasting responses, the palatable *Potamogeton pectinatus* was most supported by caging, while the distasteful *Myriophyllum spicatum* preferred open plots. Our findings may be applicable during the revitalisation of aquatic ecosystems that aims to increase macrophyte biodiversity.

## Introduction

Among plants, herbivory and competition play a key role in the formation of the plant community^1–3^. Herbivorous microfauna, mainly insects, induce the evolution of chemical defences in plants^4^, and coevolution has led to a relatively high number of foraging specialisations. Nevertheless, plant communities are also shaped by macrofaunal herbivores^2,5^ that are usually less specialised and activate both the mechanical and the chemical defences of plants. Though the effect of herbivory on terrestrial plant communities has been well studied, less attention has been focused on the impact of herbivory on communities of aquatic – particularly freshwater – macrophytes. Frequently studied factors that affect plant communities include water chemistry, sediment composition and hydro-morphological parameters^6–9^. In terms of herbivory, the impact of microfauna (i.e., crustaceans, snails and aquatic insects) on algal periphyton has been the most frequently studied topic considered thus far^10^. Nevertheless, recent studies from marine ecosystems have revealed the significant impact of herbivorous fish on macroalgae and seagrasses^11–13^. The wide retreat of kelp forests in cold areas was initiated by the climate-mediated dispersal of herbivorous fish to these localities^14–16^. An increase in temperature greater than the critical degree for cellulase-activation introduced new niches for herbivorous fish species^17^. The distribution of herbivorous freshwater fish has also recently changed; however, this change was driven by man-mediated dispersion, in addition to the natural species responses to climate changes^18–21^.

Fish and crayfish may reduce the biomass and biodiversity of macrophytes by direct consumption as well as by indirect changes in the environment^22^. The rate of herbivory affects plant growth patterns and influences the trade-off between active growth and defence^1^. Plants face a trade-off between investments into the fast growth that is necessary for success in terms of competition and into the activation of chemical defences against herbivores. This has long been well-documented in terrestrial ecosystems; however, similar findings from aquatic ecosystems have occurred relatively recently^20,23^. Observations and experiments have shown that the consumption rate of macrophytes that have a chemical defence (e.g., *Myriophyllum* sp.), is much lower than those of palatable macrophytes (e.g., *Potamogeton pectinatus*) and macroalgae (e.g., *Chara* sp.)^18,24–26^. The secondary metabolites produced by macrophytes (i.e., alkaloids, glucosinolates and polyphenolics) have an apparent impact on the feeding preferences of aquatic herbivores^27^. Nevertheless, this impact is not as well established as that observed in terrestrial plants^27,28^. In some terrestrial ecosystems, insects play an important role in herbivory, and herbivory is performed mainly by specialists^29^. However, other terrestrial ecosystems are also strongly shaped by vertebrate grazing, which is not very selective^2,5^. In aquatic ecosystems, herbivory is often performed by omnivorous species. Thus, the efficiency of macrophyte chemical defences may be lower than that of terrestrial plants^30,31^. The long-term monitoring of rudd (*Scardinius erythrophthalmus*) provided evidence about its effect on the formation of the macrophyte community^32^. A study on the plant-herbivore interactions using rudd and North American macrophytes in experimental conditions presented similar results^14^. However, another study observed no significant impact of rudd on macrophyte biomass when compared to grass carp (*Ctenopharyngodon idella*)^33^.

Disturbances that cause the partial or total removal of plant cover initiate succession^34,35^ and can result in an increase in species diversity^36^. The entire successional dynamic results from well-known trade-offs and constraints^37,38^, with the competition-colonisation trade-off  ^36,39^ being one of the most important. In terrestrial ecosystems, ruderal species dominate during the initial succession stages due to their high fecundity and ability to spread. In contrast, the best competitors are disadvantaged and cannot reach reproductive maturity due to the recurrence of disturbances; additionally, their diaspore production and dispersibility are usually low. The best competitors dominate in ecosystems where disturbances have been absent for a long time^40,41^. Though these patterns have been well-documented in terrestrial systems, it is not clear how these processes operate in aquatic ecosystems that are often insular and, thus, affected by dispersal limitations^42,43^.

It is difficult to determine the real impact of herbivory and competition on macrophyte communities in lakes. Field observations may demonstrate changes in biodiversity^44,45^ as well as in feeding preferences of herbivores^32,46^. However, without manipulation, it is impossible to disentangle the effect of individual factors.

We aimed to directly test the effect of competition among aquatic macrophytes and the effect of herbivory by manipulating both in the lake ecosystem. We utilised a cage experiment as a promising compromise to study the direct impacts of two ecological characteristics on macrophytes under natural conditions^19^. Competition was controlled by a “pulse treatment” (i.e., a treatment conducted only at the beginning^47^) including removal of the macroalgae. In addition, herbivory was controlled by a “press treatment” (i.e., a long-lasting treatment^48^) including no herbivore access during the experiment. We aimed to test the following hypotheses: (i) the decrease in competition caused by a pulse treatment will positively affect the macrophyte cover and species richness and will change the macrophyte species composition. Further, (ii) the decrease in herbivory caused by caging will also positively affect the macrophyte cover and species richness and will change the species composition. Finally, (iii) the effects of the two factors will be independent of each other.

## Results

Regardless of the treatment, all plots underwent pronounced successional dynamics that was the same as the rest of the vegetation in the lake. This was marked by the significant effect of time in all the statistical analyses. Nevertheless, the successional dynamics always changed due to the effect of the experimental treatments.

### Macrophyte cover

**Figure 1:**
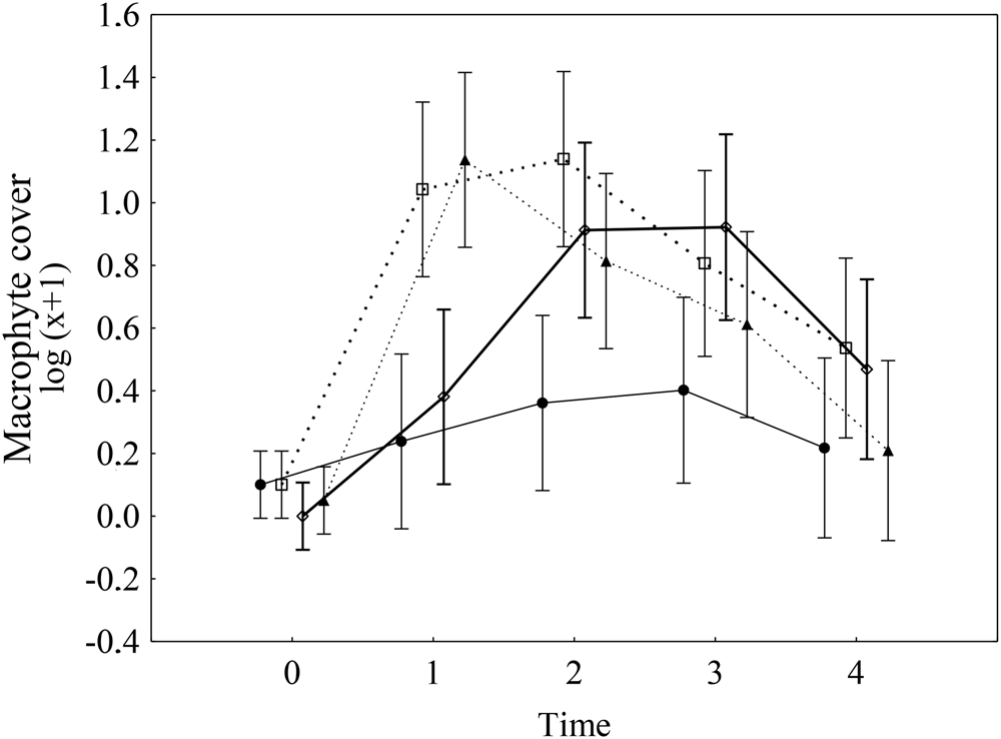
Impacts of two treatments (i.e., prevention of herbivore access and macroalgae removal) on macrophyte cover over time. Solid line = non-removal open plots (control), bold solid line = non-removal caged plots, dashed line = removal open plots, bold dashed line = removal caged plots. Data were log(x + 1)-transformed. The error bars are the 95% confidence intervals. The results of repeated measures ANOVA are in Table 1.

**Figure 2:**
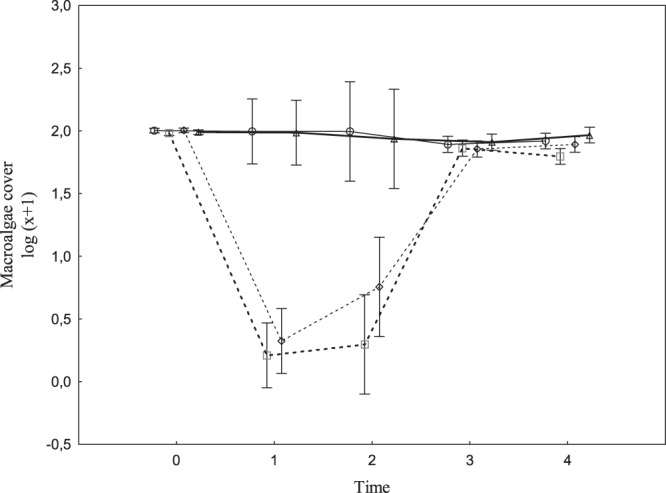
Macroalgae cover in the experimental plots over time. Solid line = non-removal open plots (control), bold solid line = non-removal caged plots, dashed line = removal open plots, bold dashed line = removal caged plots. The error bars are the 95% confidence intervals.

Hereafter, macrophyte cover refers to the cover of aquatic plants other than the manipulated dominant species of primary producers (i.e., macroalgae *Chara* sp. and *Vaucheria* sp.). Both treatments, i.e., macroalgae removal and prevention of herbivore access, had a significant positive effect on the macrophyte cover that changed over time; this included the significant main effects of both treatments and that of their interaction over time (Table 1). The increase in cover differed markedly in each treatment. Macroalgae removal resulted in an immediate significant increase in macrophyte cover during the first period (time 1; increase from 0% to 15%), followed by a gradual decrease to 1% over time (Fig. 1 and Table 2). The removal was successful, and the cover of dominant macroalgae decreased, on average, from 100% to 3% after the treatment; however, the cover gradually increased to 78% in time 4 (Fig. 2). Preventing the access of herbivores resulted in a slow gradual increase, from 0% to 11%, in macrophyte cover in time 3. The cover subsequently decreased to an average of 3% in time 4 (Fig. 1). The interaction of the two treatments was not statistically significant (neither itself, nor in the interaction with time); thus, the impacts of the two treatments on the macrophyte cover was additive on the log scale (Table 1). This might be interpreted as the two treatments having independent effects. In plots subjected to both caging and removal, the macrophyte cover increased in time 1, as it did in plots with only macroalgae removal treatment. In addition, a subsequent gradual decrease in macrophyte cover was observed, similar to the decrease seen in the cage-only plots (Fig. 1).

**Table 1:** Result of repeated measures ANOVA for macrophyte cover and species richness in time.

	DF	Macrophyte cover		Species richness	
		F	*p*	F	*p*
Removal	1	7.47	**0.01284**	4.07	0.05731
Caged	1	5.88	**0.02492**	0.68	0.41826
Removal*Caged	1	0.39	0.53730	0.01	0.92943
Error	20				
Time	4	40.12	**<10** ^**−6**^	32.69	**<10** ^**−6**^
Time*Removal	4	11.42	**<10** ^**−6**^	7.37	**0.00004**
Time*Caged	4	4.52	**0.00243**	3.06	**0.02110**
Time*Rem*Caged	4	1.19	0.32310	1.16	0.33281
Error	80				

(Time is the repeated measure factor). DF – degrees of freedom, F – value of F statistics, *p* – p values. Significant effects (*p* < 0.05) are shown in bold.

**Table 2:** Results of main effect ANOVAs (F statistics, DF = 1, 21 in all cases and corresponding *p* values) for the macrophyte cover and species richness in individual sampling dates, and test of significance of the effects on species composition in the RDA, pseudo-F values and significance obtained from the Monte Carlo permutation tests. Significant effects (*p* < 0.05) are shown in bold.

	Macrophyte cover		Species richness		Species composition	
	F	*p*	F	*p*	pseudo-F	*p*
Removal 0	0.23	0.640	0.23	0.640	0.2	0.852
Caged 0	0.23	0.640	0.23	0.640	0.2	0.845
Removal 1	**34.43**	**0.000**	**9.35**	**0.006**	**13.8**	**0.000**
Caged 1	0.03	0.859	0.02	0.898	1.0	0.391
Removal 2	**6.52**	**0.019**	**10.94**	**0.003**	**3.8**	**0.021**
Caged 2	**10.85**	**0.003**	3.26	0.085	**13.6**	**0.000**
Removal 3	0.11	0.747	1.48	0.238	0.3	0.859
Caged 3	**6.24**	**0.021**	0.20	0.659	**14.2**	**0.000**
Removal 4	0.05	0.828	1.83	0.191	<0.1	0.949
Caged 4	**4.62**	**0.043**	**7.73**	**0.011**	**15.7**	**0.000**

**Figure 3:**
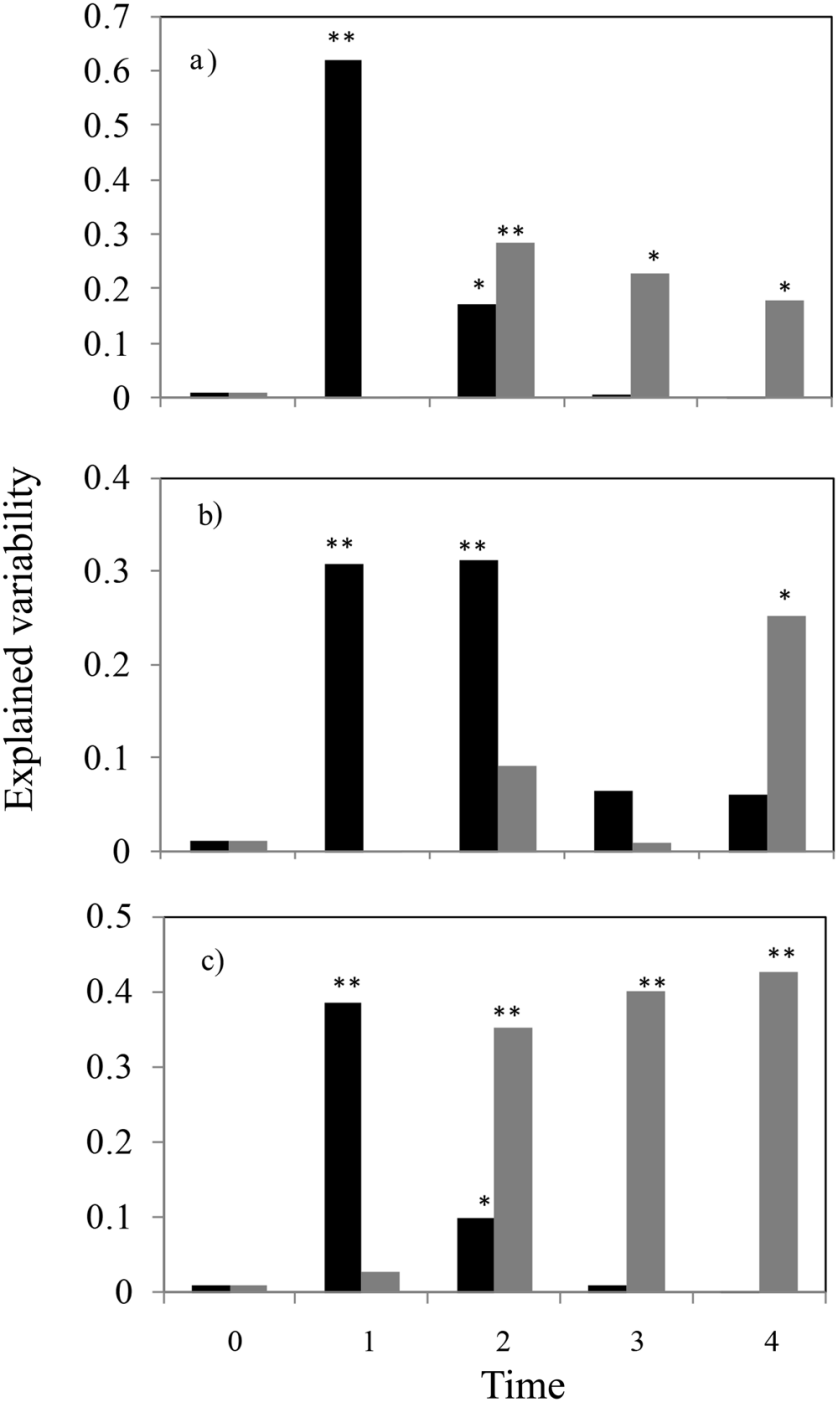
Proportion of total variability explained by two treatments, i.e. by macroalgae removal (black columns) and by prevention of herbivore access (grey columns) as main effects in ANOVA of (**a**) macrophyte cover and (**b**) species richness, and (**c**) in RDA of quantitative species composition calculated separately for each time interval (0–4). One and two asterisks show the significance of individual effects (*p* < 0.05 and *p* < 0.01, respectively, obtained from corresponding F-tests of the main effects in ANOVA in (**a**,**b**), and by Monte Carlo permutation test in (**c**). Detailed results of individual ANOVAs are in Table 2, and individual ordination diagrams are in Supplementary Fig. S1.

In the control plots, the changes were the least evident, and the macrophyte cover was the lowest (Fig. 1, Table 2). The explained variability in ANOVA showed that, macroalgae removal initially had a larger effect than did the prevention of herbivore access (Fig. 3a); however, the effects of macroalgae removal quickly faded. The decrease in the effect was apparently caused by the return of macroalgae to the experimental plots (Fig. 2). The prevention of herbivore access reached its maximum effect in time 2, and then the effect slowly decreased but remained significant until the end of the experiment. In contrast, the highest effect of the macroalgae removal was measured in time 1 and then decreased quickly (Fig. 3a).

### Species richness

**Figure 4:**
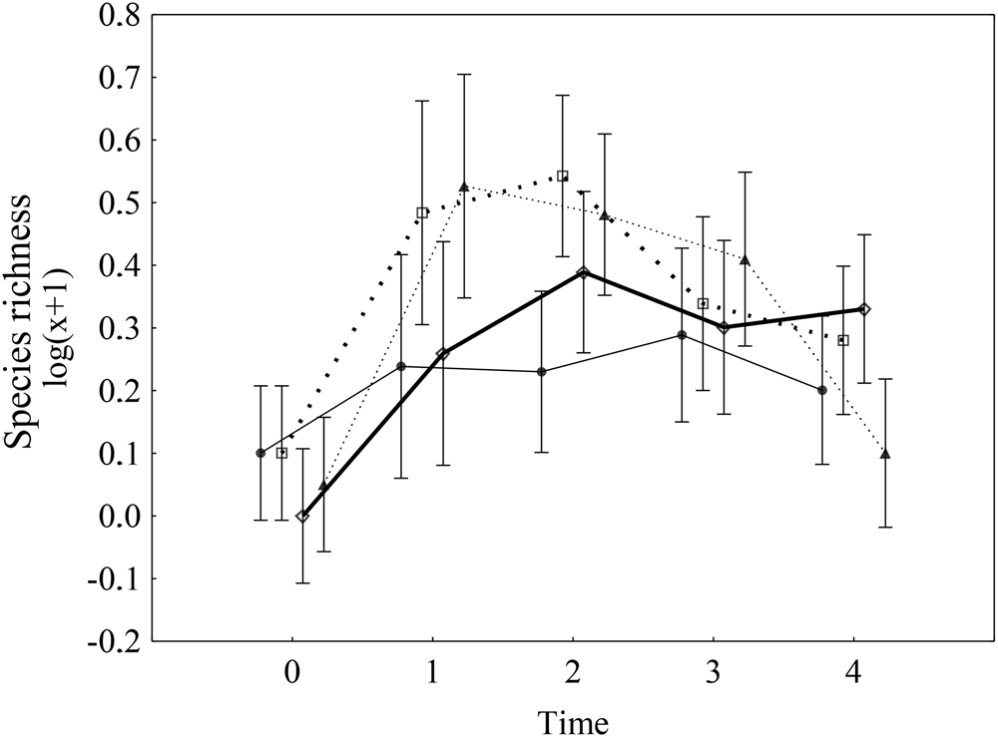
Impacts of two treatments (i.e., prevention of herbivore access and macroalgae removal) on macrophyte species richness over time. Solid line = non-removal open plots (control), bold solid line = non-removal caged plots, dashed line = removal open plots, bold dashed line = removal caged plots. Data were log(x + 1)-transformed. The error bars are the 95% confidence intervals. The results of repeated measures ANOVA are in Table 1.

As with macrophyte cover, species richness was positively affected by both treatments (i.e., both had a statistically significant interaction with time), and their interaction was not significant, neither by itself, nor in its interaction with time (Table 1, Fig. 4). In addition, similar to macrophyte cover, the effect of removal was very pronounced at the beginning of the experiment, but this effect disappeared in the last two observations; in contrast, the effect of caging was rather negligible at the beginning of the experiment but appeared as significant in the last observation (Fig. 3b). In the case of macroalgae removal, species richness increased from one species in time 0 to five species in time 1 (each species was present in at least one of six plots for each treatment), and species richness subsequently decreased to one species per plot in time 4. The prevention of herbivore access resulted in a gradual increase in species richness, followed by a more steady-state (Fig. 4). The interaction of the two effects was not significant (Table 1). The lowest species richness was generally observed in the control plots (Fig. 4).

### Species composition

**Figure 5:**
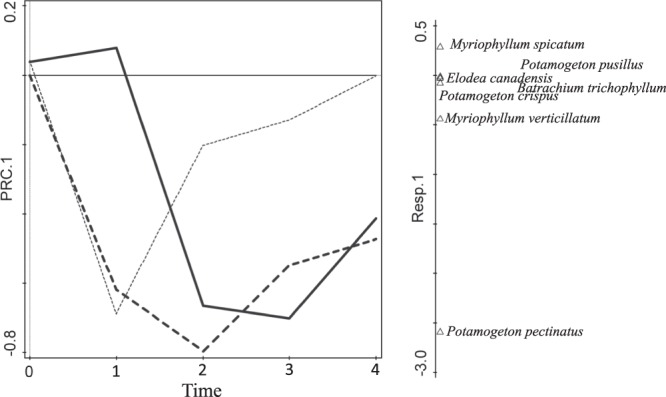
Response of the community to experimental manipulations visualised using principal response curves (PRC). The horizontal axis (time) corresponds to individual observations, with 0 being the baseline (just before the treatments were imposed; the variation in the baseline provides the size of the expected variation in plots under the same treatment). The x-axis (light solid line) corresponds to the reference group (we deliberately selected the non-removal open as the control), bold solid line = non-removal caged plots, dashed line = removal open plots and bold dashed line = removal caged plots. The Resp. 1 axis on the right shows the species score on the first PRC axis and is aimed to help interpret the response (e.g., negative values of the PRC.1 correspond to prevalence of *Potamogeton pectinatus*).

In total, seven macrophyte species and two macroalgae species were observed in the experimental plots. The species composition showed pronounced dynamics, i.e., both common successional dynamics and differential responses to the treatments. The first PRC (principal response curves) axis (Fig. 5) was determined by the two most common species and their differential responses to caging; in contrast, *P. pectinatus* was strongly supported by the exclusion of herbivores, and *Myriophyllum spicatum* preferred the open plots. The PRC diagram (highly significant, pseudoF = 52.3, *p* = 0.0002, Fig. 5) clearly demonstrated the responses to experimental manipulations. While the response to macroalgae removal was immediate and pronounced, the effects vanished over time, as can be expected for a “pulse type” treatment; however, the effects of caging were gradual but rather long-lasting, corresponding to the “press type” treatment. This also corresponds to the amount of explained variability in individual RDA for the individual times (Fig. 3c, Table 2).

At the beginning of the experiment (i.e., time 1), the community composition was determined mainly by macroalgae removal (Fig. 5: the PRC curves in time 1 differ for removal, and in the RDA results for time 1, Supplementary Fig. S1, the first axis was determined by removal); additionally, most species responded positively to removal (with the exception of *Potamogeton crispus*). At time 2, caging was more important (the first RDA axis for times 2–4 were determined by caging). However, in time 2, both the effects were significant, and we can see a predominance of most of the species, i.e., *M. spicatum, Myriophyllum verticillatum, Elodea canadensis* and *P. crispus* predominated in the plots where macroalgae were removed. Interestingly, *P. pectinatus* responded positively to caging beginning in time 2, whereas the predominance of *M. spicatum* in the open plots started only in time 3. Only two species of macrophytes occurred in plots with no treatment: *M. spicatum* and *P. pectinatus*. Three species occurred in plots with prevention of herbivore access: *M. spicatum*, *P. pectinatus* and *P. crispus*. These three species, plus *M. verticillatum, E. canadensis* and *Batrachium trichophyllum*, occurred in plots where macroalgae were removed. In plots with both treatments, all mentioned species except *E. canadensis* were observed, and additionally, *Potamogeton pusillus* appeared.

## Discussion

Our study demonstrated how competition and herbivory affected the structure of the aquatic ecosystem in terms of affecting the macrophyte cover, species richness and composition of macrophytes. The results supported our hypothesis that the pulse treatment (i.e., macroalgae removal) would result in a marked increase in the macrophyte cover and macrophyte species richness in the experimental plots; however, the initial effects quickly diminished and finally disappeared. The increase was apparent mainly during the first time period (i.e., time 1). Subsequently, the macrophyte cover decreased because macrophytes were gradually replaced by macroalgae. The species richness also started to decrease in time 1. A similar trend was observed during the succession stages of a drying lake in France, where the greatest species richness was observed during the first year^42^. A lake, like an island, is a semi-isolated ecosystem with a limited possibility of species dispersal from the outside^49^. Thus, a few competitively successful species may colonise the disturbed habitat after a short time, resulting in highest species richness occurrence during the initial succession stage^50^; however, species richness subsequently decreases due to competitive exclusion. Macrophyte species that occurred only in plots after the pulse treatment – macroalgae removal (i.e., after the elimination of competition) included *M. verticillatum*, *E. canadensis* and *B. trichophyllum*. These three species were scarce, and each of them accounted for less than 1% of the total vegetative cover in the lake^17^. In addition, *M*. *verticillatum* is included on the IUNC red list and is classified as vulnerable (A2c). Although *E. canadensis* is an invasive species and is spreading worldwide^51^, the conditions in Milada Lake, such as trophy, water chemistry, sediment composition and hydro-morphological parameters^6–8^, are probably sub-optimal for its growth. The next species, *B. trichophyllum*, was observed in Milada Lake for the first time. Its occurrence demonstrated the apparent impact of disturbances on the community of primary producers in the lake. In terms of the condition of our study site, all the species mentioned above represent species that have the ability to quickly colonise and have low competitive ability^36^. Considering the isolation of Milada Lake, we may exclude hydrochory (i.e., water dispersal through ditch networks) in terms of colonisation by the new species *B. trichophyllum*. We assumed that waterfowl may have had an important impact on the dispersal of macrophytes to our locality, but we have no clear evidence to support our theory. Nevertheless, this hypothesis has been generally accepted^52–54^. Specifically, internal dispersal, which refers to the eating and excretion of seeds by waterfowl, has a more important role than does external dispersal, which refers to seeds that have attached to the body surface, a process that has been overestimated in the past^55^. However, we admit that the species could have already been in the lake, but if so, it was present only at very low densities that were not recorded during our regular monitoring.

Our hypothesis that the decrease in herbivory caused by caging will positively affect both macrophyte cover and species richness was confirmed. The effect appeared much later and was particularly pronounced in terms of species composition, which increased with time. The results support the latest findings that herbivory in aquatic ecosystems is noticeable. Until the 1990s, herbivory on aquatic vascular plants was considered relatively unimportant; however, new findings show that 48% of plant biomass may be removed due to herbivory. This is, in fact, a pressure that is five to ten times greater than that reported in terrestrial ecosystems^56^. According to Wood *et al*.^57^, changes in plant abundances were reported at relatively low herbivore densities, suggesting that, in aquatic systems, greater herbivore densities overwhelm plant compensatory growth responses. The more intensive utilisation of plant biomass in aquatic ecosystems is probably due to low C: N ratio (i.e., the high content of nitrogen) in aquatic ecosystems^56^. We focused on the level of impact of herbivory on the macrophyte cover and species richness. Our complex monitoring of fish populations at the study site showed that the abundance of potential herbivorous fish able to affect the macrophyte community was 9–15 kg ha^−1^ (J. Peterka *et al*., unpubl. data). Rudd has the greatest impact in the studied lake^17,58^. Thus, rudd poses the most herbivorous pressure on the macrophytes in this experiment. Other potential herbivores include roach (*Rutilus rutilus*)^58^ and spiny-cheek crayfish (*Orconectes limosus*; based on our observations), which are abundant in the lake but present a lower impact. We may exclude the impact of herbivorous waterfowl^52^, particularly the mute swan (*Cygnus olor*) and common coot (*Fulica atra*), because the experiment occurred at the depth of 3–4 m^59^. In addition, a recent meta-analysis by Wood *et al*.^57^. referred to substantial between-taxa differences in the effects of herbivores on the abundance of freshwater and marine macrophytes. Fish have large impacts on macrophytes, while insects and birds have relatively low impacts on macrophytes. The reason for these differences may be the mobility and habitat preferences of each of these groups. Fully aquatic species, which permanently live underwater, have been shown to produce the greatest impacts on aquatic plants and are often considered to be ecosystem engineers^60^. Two species from our herbivores were invasive species in certain parts of the world, e.g., the spiny-cheek crayfish in Europe^61^ and rudd in North America and New Zealand^18,20^. Therefore, it is advisable to have knowledge about their structuring role in an aquatic ecosystem.

The decrease of herbivory caused by caging also affected the species composition. This result is in accordance with the theory that herbivory has an important impact on the succession of macrophytes^56^. At our study site, the highest expansion was recorded for *P. pectinatus*, while the lowest cover was recorded for *M. spicatum*. These two species were the most common macrophytes in the experimental area as well as in the entire lake. The results clearly showed a preference for *P. pectinatus* by herbivores, but *M. spicatum* was ignored; furthermore, *M. spicatum* predominated in the open plots. The predominance of *P. pectinatus* in the caged plots appeared earlier than did the predominance of *M. spicatum* in the open plots. This suggested that *M. spicatum*, as a competitively weak species, was resistant to herbivory and took advantage of the open plots that suppressed the other species, particularly *P. pectinatus*. This result is in agreement with previous studies that showed that *P. pectinatus* was readily utilised by rudd^17,20,23^. The distaste for *M. spicatum* was probably caused by their high phenolic concentration, which is used as a chemical defence against herbivory^23^. The low number of *M. spicatum* in closed plots was probably due to its low competitiveness compared to *P. pectinatus* and other macroalgae that are more successful when herbivory is absent. This result demonstrates a typical trade-off, i.e., species with a chemical defence against herbivory are bad competitors and vice versa^1^. Like *P. pectinatus*, *P*. *crispus* was also positively affected by the “prevention of herbivore access” treatment but in considerably lower abundances. This result is in accordance with the claim that *Potamogeton* species generally represent a genus preferred by herbivores^17,20,32^, probably due to their low chemical defences^23^. *P. crispus* tries to defend itself with tough and partially spine-like leaves, but it seems that this type of defence is not very effective.

We found no significant interaction between the two treatments; thus, we can conclude that the effects of herbivory and competition were independent in our case (as no interaction means additivity on the log scale, for both species richness and macrophyte cover; additionally, the caging means the same percentage increase in both variables in the control and removal plots, and removal means the same percentage increase in the caged and open plots). Nevertheless, a positive effect of both treatments together was recorded for *P. pusillus* and *B. trichophyllum*. Both species were rare in the experiment as well as in the entire study lake. This indicated favoured utilisation by herbivores (results for similar species in^20,32^) and highlighted their low competitiveness due to the suboptimal conditions in the lake, which was mainly represented by low nutrient availability^6,7,10^. However, due to the scarcity of these two species, they had very small effect on the results of the statistical tests.

We have clearly demonstrated that both herbivory and competition were strong ecological forces that shaped the macrophyte community composition. The results of our study may also be applied in revitalisation or conservation projects conducted in aquatic ecosystems. The regular disturbance of a lake bottom in a restricted area covered by macrophytes may lead to an increase in the biodiversity and preservation of rare species in the community, either by the colonisation of the uncovered bottom or by strengthening the diaspore production in the system^42,62^. A similar effect has been observed in various terrestrial ecosystems^63^. In contrast, human-induced disturbances of seagrasses in marine ecosystems may have a negative impact on sensitive species^64^. Thus, the controlled disturbances should be evaluated individually. Our results implied that disturbances such as those studied in our experiment will increase the biodiversity in an aquatic ecosystem. However, this approach cannot be applied without precautionary measures, as not all species would benefit from similar interventions. The treatment should be mainly performed in a limited area and should be continued after the pilot data have been evaluated. Though it is well-known that the species composition and quantity of the fish community affect the water quality^65^, we have newly demonstrated that vertebrate herbivory can decrease the quantity and species richness of aquatic macrophyte communities. We have also demonstrated the effect of macroalgae competition on macrophytes. Thus, management that decreases these effects might lead to the increase in biodiversity and may even promote some endangered macrophytes species.

## Methods

### Study site

**Figure 6:**
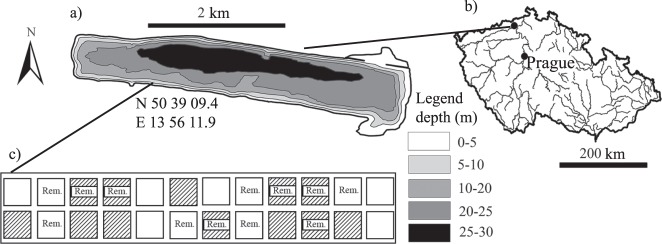
Study site. (**a**) Bathymetric map of Milada Lake, with the location of the experiment. (**b**) Map of the Czech Republic, showing location of the lake. Both maps were generated by the software ArcMap, version 10.2.2^69^. (**c**) Schematic design of the experiment: 24 experimental plots with different treatments. White and patterned squares represent open and closed cages, respectively. *Rem* stands for the macroalgae removal.

The study was conducted in the newly created, opencast mine Milada Lake (50°39′N, 13°58′E; Fig. 6a) in the Czech Republic (Fig. 6b). Milada is oligo- to meso-trophic with mean summer total phosphorus in surface layer (TP) <10 µg L^−1^. The lake has an area of 250 ha, a volume of 0.036 km³ and a maximum depth of 25 m. Aquatic restoration started in 2001 (when the water level was 122 m.a.s.l.), and the final water level (i.e., 145.7 m.a.s.l.) was obtained in 2011. Several species of macrophytes and macroalgae are present at high biomasses to a depth of 12 m^17^.

### Experimental design and sampling

The experiment was conducted in the southwest part of Milada Lake (N: 50°39′09.4″, E: 13°56′11.9″) at a depth of 3–4 m (i.e., the depth with the highest macrophyte biodiversity; I. Vejříková, unpubl. data) and started in May 2014. Experimental plots were delineated by cages made of stainless steel with a width of 2 × 2 m and a height of 1.6 m. The optimal sizes were chosen based on the sizes of potential herbivores^66^ and the accuracy of cover estimates that generally decreases with the size of the plot. Two treatments were applied: (i) the removal of macroalgae *Chara* sp. and *Vaucheria* sp. (i.e., the pulse treatment), and (ii) the prevention of herbivore access (i.e., the press treatment) by using closed cages made of a stainless steel net with mesh sizes of 20 mm (the controls were open cages). A full factorial design formed four combinations when either the first treatment, the second treatment, both treatments, or no treatment was applied. Each combination was replicated six times, and the plots were completely randomised (with restrictions to prevent the clumping of identical treatments in space; Fig. 6c). We also attempted a third treatment, i.e., transplanting two bunches of *M. spicatum* and two bunches of *P. crispus* to the plots, but the plants did not establish (i.e., transplants were seen dead in time 1); thus, this third treatment was disregarded. The potential herbivores were rudd, roach and spiny-cheek crayfish. The abundance and biomass of rudd was 7 ind. ha^−1^ and 3 kg ha^−1^, respectively, in 2014, and 91 ind. ha^−1^ and 7 kg ha^−1^, respectively, in 2015. The abundance and biomass of roach was 149 ind. ha^−1^ and 12 kg ha^−1^, respectively, in 2014, and 14 ind. ha^−1^ and 2 kg ha^−1^, respectively, in 2015 (J. Peterka *et al*., unpubl. data). Both fish species were concentrated in the littoral section of the lake, which also has high macrophyte occurrence (based on our observations). The plots were initially sampled before the experimental treatment and then four times after the treatment, i.e., two samples in July and September in both 2014 and 2015, respectively. The cover of all aquatic plants and algae was visually estimated by two independent SCUBA divers in each plot. The plot was visually divided into four 1 × 1-m subunits, and the cover was estimated in given subunits and averaged by each diver; finally, the mean cover obtained from both divers was used as the final value. The estimation of the macrophyte cover varied between the divers by 1.8% (from 0 to 8%). The water transparency varied from 3 to 6 m during the experiment. To prevent potential shading, the microalgae and sediments were regularly removed by a small broom from the cages, particularly from the lid of the closed cages.

### Statistical analysis

The macrophyte community was characterised by species richness, macrophyte cover (univariate response variables), and species composition. The univariate variables (i.e., species richness, macrophyte cover and macroalgae cover) were analysed using repeated measures ANOVA. The data were log(x + 1)-transformed before analysis to improve normality and homoscedasticity. Because the effect of both treatments changed over time, we conducted separate analyses (i.e., separate two-way ANOVAs) for each individual observation. We also presented the amount of explained variability by each of the main effects at each time point (the amount of explained variability was calculated as SS_effect_/SS_total_ for each treatment). Because it was a manipulative experiment, the explained variability can be considered a measure of strength of the effect of manipulation, and the statistical significance is proof of causality. The univariate analyses were conducted using Statistica 12 (StatSoft Inc.).

The species composition was analysed in the framework of constrained ordinations. Because the data were in the form of repeated observations, we used PRC^67,68^. Because the PRC (highly significant) demonstrated that the effects of the two treatments changed considerably over time (similar to the univariate analyses), we used simple analyses of the effects of the two treatments (i.e., removal and caging) from individual observation times. In this case, variation partitioning^68^ was used to quantify the independent effects of the two factors and to test for their significance. Because the two factors were orthogonal, the overlap of their two effects was, by definition, zero; thus, we were only interested in the size of the two effects. This procedure provided a multivariate equivalent of the ANOVA results.

The multivariate analyses were conducted using CANOCO5, and the significance of the effects was obtained using Monte Carlo permutation tests (in PRC, with a corresponding hierarchical permutation scheme), with 4999 permutations.

## Electronic supplementary material

Supplementary Information

## Data Availability

The dataset analysed during the current study is available from the corresponding author on reasonable request.
