# Supplementary material for: Impact of herbivory and competition on lake ecosystem structure: underwater experimental manipulation
Source: Sci Rep. 2018 Aug 14;8:12130. doi: 10.1038/s41598-018-30598-0 (PMC6092342; doi:10.1038/s41598-018-30598-0)
Supplement: Supplementary file 1 — Supplementary Information [file 41598_2018_30598_MOESM1_ESM.pdf]

Supplementary information for manuscript:

Impact of herbivory and competition on lake ecosystem structure: underwater experimental manipulation

Ivana Vejříková, Lukáš Vejřík, Jan Lepš, Luboš Kočvara, Zuzana Sajdlová, Martina Čtvrtlíková, Jiří Peterka

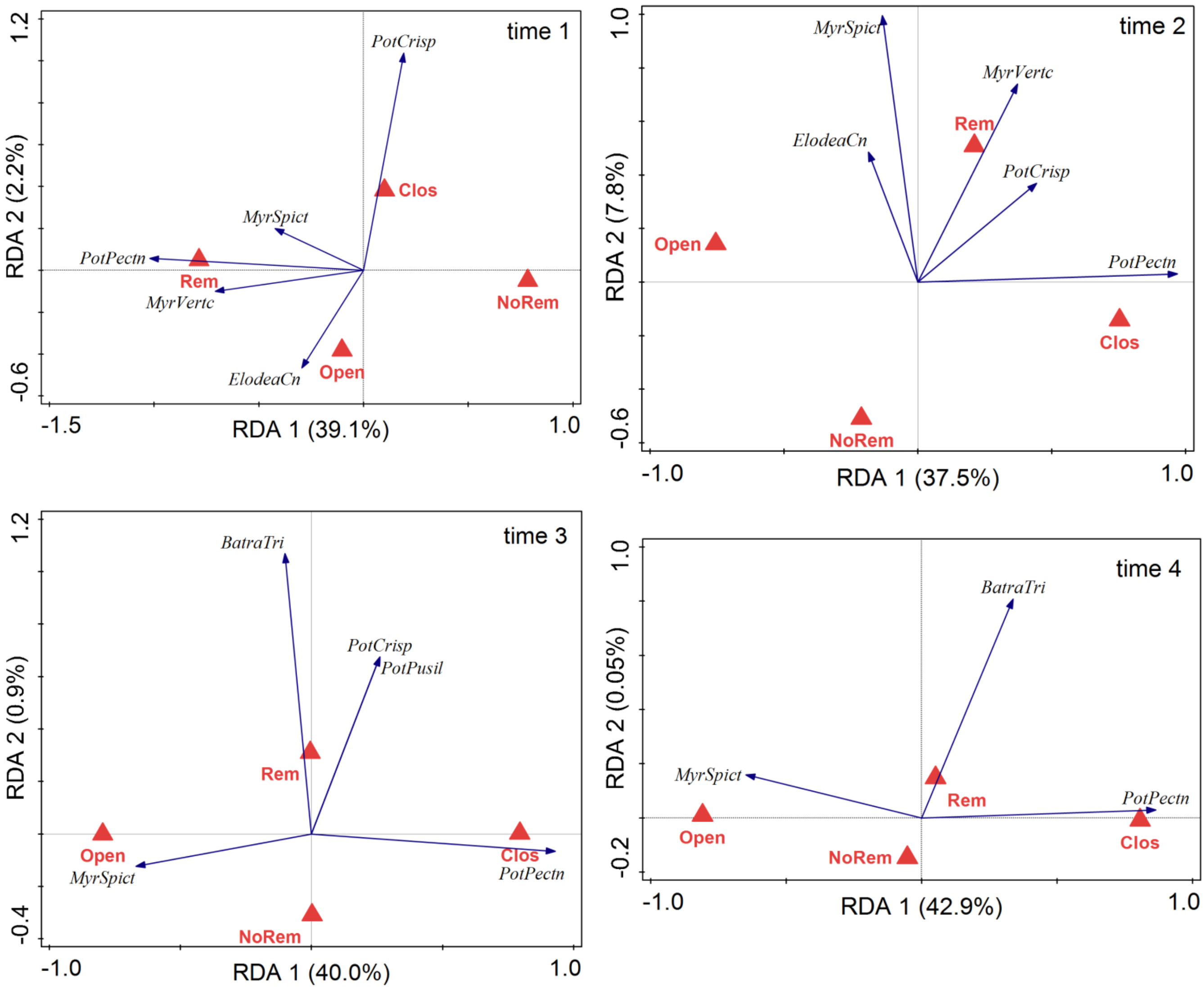

Figure S1. Ordination diagrams of the RDA analyses for individual times illustrate the species preferences for individual treatments. Open and Clos(ed) represent plots that were accessible or inaccessible for herbivores, respectively. Rem and NoRem represent plots where macroalgae were or were not removed , respectively. The values accompanying the axes are percentages of total variability in species composition explained by given RDA axis. The symmetric scaling (i.e., With the same focus on interspecies correlation and on intersample (and thus also intertreatment) distances was applied. No effect was significant in the baseline data (time 0) and thus the ordination diagram is not displayed. The detailed statistical results (significance of individual factors) are in Table 2.
